# Lightweight Security-by-Design for TinyML Models in Constrained Embedded Environments

**DOI:** 10.3390/s26185727

**Published:** 2026-09-09

**Authors:** Haifa A. Alanazi, Khaznah R. Alshammari

**Affiliations:** 1 Department of Information Systems, College of Computing and Information Technology, Northern Border University, Rafha 91911, Saudi Arabia; 2 Department of Computer Science, College of Computing and Information Technology, Northern Border University, Rafha 91911, Saudi Arabia; khaznah.alshammari@nbu.edu.sa

**Keywords:** TinyML, security-by-design, model compression, adversarial robustness, model extractionl, lightweight cryptography

## Abstract

TinyML models now run inference directly on microcontrollers with only kilobytes of memory, inside sensors, wearables, and industrial devices that often have no cloud link and no way to receive a patch once deployed. This makes them an easy target, since compressed models resist adversarial and extraction attacks less than full-precision models, but most TinyML research still treats security as a step added after compression rather than during it. This paper proposes a three-layer security-by-design architecture built to close that gap. The first layer scores candidate compressed models on a joint fitness function that combines clean accuracy, adversarial robustness, and model size before the final architecture is fixed. The second layer signs and verifies model weights with the lightweight ASCON cipher at boot and during over-the-air updates. The third layer is a runtime monitor that computes a live distribution-drift score on incoming queries and raises local defense strength once that score crosses a fixed threshold, without needing a cloud round trip. The full pipeline was validated on real microcontroller hardware, not simulation, and stays inside a 62 KB RAM budget with inference latency under 10 ms. Against real baseline models on Edge-IIoTset, the proposed method reaches 94.3% accuracy, the highest of the models tested, while cutting attack success rate from over 60–84% down to 38.9% and raising the query cost for model extraction more than five times over the weakest baseline. An ablation study and a five-seed statistical test confirm the gain holds and comes from the combination of all three layers, not from any single part.

## 1. Introduction

TinyML has moved machine learning inference out of the cloud and onto microcontrollers with only tens or hundreds of kilobytes of memory [1]. These devices now sit inside industrial sensors, wearables, and agricultural monitors, running classification and detection tasks locally so the device does not need a constant network link. The nodes this paper targets are physical sensing units, temperature, pressure, and flow sensors among them, that run their own classification model on the sensor stream rather than forwarding raw readings to a gateway; Section 3.1.1 lays out this sensor-to-MCU-to-cloud architecture in full. This shift cuts latency and power draw, but it also changes the security picture in a way most published work has not caught up with. A cloud-hosted model can be patched the moment a flaw is found. A model flashed onto a microcontroller in a remote field installation often cannot be reached again for months, so whatever defense ships with it at build time is the only defense it will ever get.

There is no question this is a real risk, as recent work has shown. Adversarial examples generated on a powerful host will continue to deceive models even after they are ported to actual boards like the ESP32 or Raspberry Pi [2]; the threat does not dissipate when one leaves the lab for the field. Then there are compressed models. Quantization and pruning strip away the very redundancy that makes a full-precision network difficult to fool, leaving them more open to side-channel and adversarial attacks [3,4]. An attacker in possession of the device has a physical attack surface to exploit as well: without the kind of key-refresh mechanism found in cryptographic hardware, embedded neural networks can be probed for model parameters via power and timing [5].

Yet security is an afterthought in much of the literature. A systematic review puts the number of TinyML papers that give it any consideration at under 5%, with even fewer dealing with the particular threats of TinyML as opposed to IoT in general [3]. The regulatory environment is changing, however. Under the EU Cyber Resilience Act, an unverified model update is considered a security flaw tantamount to a firmware bug; the law now mandates secure boot, signed firmware and updates, and a software bill of materials [6].

What one sees across these studies is a matter of priorities. Security is put to the test only after compression has been optimized for size and latency, if it is tested at all. By then the quantization scheme and architecture are set, so a robustness test has little opportunity to remedy what it uncovers. Moreover, extraction defenses and adversarial robustness tend to be treated as distinct issues in separate publications, never as a unified design problem on the same hardware. This paper is meant to bridge that divide. We have developed a three-layer architecture for real MCU hardware that does not look at things in isolation; each layer is set relative to the other two rather than tuned on its own, so the design is a coupled system rather than three independent tools placed side by side. A joint fitness function for robustness and size steers the compression process, a light cipher is in place to safeguard the model at rest and during updates, and a runtime monitor adjusts its defenses according to the behavior of live queries. The contributions of this paper are:

- A security-aware compression search, formalized as a joint fitness function over clean accuracy, adversarial robustness, and size, that filters weak candidates before the final compressed model is fixed.
- A lightweight cryptographic wrapper using ASCON [7] to sign and verify model weights at boot and during over-the-air updates, addressing the integrity requirement raised by the EU Cyber Resilience Act [6].
- An adaptive runtime monitor, driven by a Kullback–Leibler divergence score over query windows, that raises local defense strength when drift crosses a fixed threshold, without a cloud round trip.
- A joint evaluation across accuracy, resource cost, energy, and attack resistance on real MCU hardware, including a statistical significance test across repeated seeds.
- An ablation study that isolates the contribution of each layer, showing the security gain comes from the combination of all three, not from any single part.

We have structured the remainder of the paper in this manner. A survey of current TinyML security research is provided in Section 2, along with an assessment of its deficiencies. The methods are laid out in Section 3, which details the dataset and preprocessing steps as well as the three-layer architecture, including the relevant algorithms and equations. In Section 4 we present our findings vis-à-vis real baseline models on matters of classification, robustness, energy expenditure and statistical significance. Section 5 puts those results in context and notes any design limitations. Finally, the principal conclusions and an outlook on future work are offered in Section 6.

## 2. Related Work

### 2.1. Adversarial and Side-Channel Threats on Constrained Devices

Shah et al. [2] tested adversarial attack transferability directly on ESP32 and Raspberry Pi hardware and found that perturbations crafted on a powerful host machine still fool the on-device model, which confirms the threat holds once a model actually leaves the lab. Silva et al. [8] ran a matched attack-and-defense study on quantized neural networks placed at the deep edge, borrowing models and tasks from the MLPerf Tiny benchmark suite [9], but their evaluation stays limited to vision tasks and does not cover extraction or side-channel threats. Preuveneers et al. [4] looked specifically at how full-integer quantization changes adversarial robustness at the edge and found the effect is not uniform across bit-widths, which is part of the motivation for scoring robustness during the compression search itself rather than after it, as done in Layer 1 of this paper. Maji et al. [5] showed that embedded neural network implementations leak enough information through power and timing side channels to recover both architecture and weight values, and pointed out that ML inference lacks the key-refresh mechanism that limits how many traces an attacker can capture against a cryptographic implementation. Paul and Zuzak [10] build on this power-channel angle from the defender’s side, using correlational power analysis to flag tampered model weights on an STM32 board at runtime, but their method checks integrity only; it does not touch adversarial robustness or extraction resistance, and it needs a golden reference signature rather than working from the compression search itself. A few papers do move the defense at runtime instead of fixing it at build time. Song et al. [11] switch between a pool of vision models on an embedded platform to defeat adversarial examples, and Qian et al. [12] apply a similar moving-target idea to edge intelligence, changing which model answers a query. Both show that runtime variation raises the cost of an adversarial attack, but the model pool itself is fixed and chosen at build time, and neither work touches extraction resistance or key management on the same device. A broader survey [3] builds a formal threat taxonomy spanning physical and remote attack vectors specific to microcontroller-class devices, separating TinyML from generic IoT and EdgeML security work, but this survey stays a mapping exercise. It does not propose or test a defense architecture of its own.

### 2.2. Model Extraction, Cryptographic Defense, and Detection Datasets

The 2025 survey on model extraction categorizes side-channel leakage as an attack family in full, and also states that edge devices under resource constraints tend to run compressed models, which are easy to clone once they allow repeated queries [13]. On the side of model extraction defenses, Chakraborty et al. [14] propose a method that changes the way of the query responses depending on the suspicion score at runtime, rather than relying on a single policy. The method aims at a cloud-based MLaaS API with a stable network connection, not a disconnected MCU-class device, and does not provide any integrity protection for the model. On the side of cryptographic protections, NIST announced ASCON as a lightweight cryptography standard for constrained devices [7]; the cipher is designed to fit into a couple of kilobytes of RAM, much less than AES-GCM requires on the same hardware. Baciu et al. [15] use a similar but distinct approach to establish boot time trust, using a combination of a device-identity token and a model-identity token so that a relying party can authenticate both the board and the model that runs on it; this addresses the integrity identity and authorization problem fairly well, but relies on the existence of a reachable relying party, something which is not the case with the disconnected field-deployment targeted in this paper, and fails to address anything once the model is up and running; there is also no cited approach which couples a token-exchange-based scheme with ASCON or another lightweight cipher with an adaptive, model-level defensive layer. In terms of detection performance, IIoT-TinyDNN [16] achieves an accuracy of 92.99% on Edge-IIoTset [17] with only 2255 parameters, but offers no form of defense whatsoever, sacrificing security in favor of model size. A lightweight, energy-efficient IDS [18] achieves an F1-score of 0.94 on all three sets—Edge-IIoTset, CICIoT2023 [19] and RT-IoT2022 [20] —but requires 120–180 ms per input on a Raspberry Pi 4, failing to be used in real time on MCU-like devices. The defense techniques that are aimed at quantization models, such as TriQDef [21], decrease the effectiveness of patch attacks by more than 40 percent for quantized neural networks, yet their efficiency was shown on datasets such as CIFAR-10 and ImageNet instead of a restricted IoT intrusion dataset and were not combined with any cryptographically secure layer.

### 2.3. Privacy-Preserving Learning on Heterogeneous and Constrained Systems

A related line of work protects the training data rather than the deployed model. Wu et al. [22] combine an adaptive gradient-descent scheme with differential privacy for federated learning, so participants in a multi-party training round keep their local data private while the shared model still converges under a fixed communication budget. Wang et al. [23] apply a similar differential-privacy mechanism to deep-learning-based content caching in heterogeneous communication networks, perturbing client-side signals before they are used for caching decisions. Both papers confirm that privacy-preserving mechanisms can be made to fit constrained and heterogeneous network settings without breaking model utility, which supports the general premise of this paper; security and constrained deployment are not mutually exclusive, but neither addresses the threats this paper is concerned with: once training is done and a compressed model is sitting on a microcontroller, differential privacy does not stop an adversary from perturbing inputs to force misclassification, tampering with the flashed weights, or cloning the model through repeated queries.

### 2.4. Research Gap

Three patterns repeat across this body of work. Security is checked after compression, not folded into the compression search itself. Each paper targets one attack family, adversarial, extraction, side-channel, or integrity at boot, rather than treating them as one combined threat model on the same device. A few recent defenses do adapt behavior at runtime, switching between a fixed pool of models [11,12] or adjusting query response on a cloud API [14], but none of this runtime adaptation reaches a disconnected MCU-class device running a single compressed model, and none pairs it with build-time integrity checking. To the best of our knowledge, no published defense for constrained embedded devices combines a live drift signal, adaptive defense strength, and a cryptographic integrity layer in one design. This paper’s three-layer design answers all three points together, tested end to end on real MCU hardware rather than through separate, disconnected experiments.

## 3. Materials and Methods

### 3.1. Dataset and Preprocessing

This work uses Edge-IIoTset [17] as the primary dataset, since it covers fourteen attack types across five categories, DoS/DDoS, information gathering, man-in-the-middle, injection, and malware, collected from real IoT and IIoT devices including temperature sensors, pH meters, and heart rate monitors. The raw capture carries more than one thousand features. After feature pruning, sixty-one features remain, since most raw fields add noise rather than signal on a device with a strict memory budget. Cross-dataset generalization is tested separately on CICIoT2023 [19] and RT-IoT2022 [20], without retraining, to check whether performance holds outside the dataset used for training. A custom attack trace set is also built on physical hardware, following the transferability protocol used on ESP32 and Raspberry Pi in [2], where adversarial and extraction queries are logged directly from an ESP32 and an STM32 board rather than simulated on a host machine.

#### 3.1.1. Sensor-Node Deployment Architecture

Since this work targets a sensor node rather than a generic embedded classifier, the physical setup is described here before the preprocessing steps. The protected node is an IIoT sensor node of field IIoT sensors of types temperature, pressure, or flow (compatible with the existing device classes of Edge-IIoTset) that emits unprocessed readings into a local MCU using a wired serial or I2C communication channel. The MCU (STML476RG, with Cortex-M4 processor, to perform the crypto and monitor layer functionality, and ESP32-WROOM-32, with dual-core Xtensa LX6 to run the wireless variant) performs inference directly on the sensor data, thus performing inference at the edge node rather than on a gateway node. The update channel is the OTA communication channel and is activated only when a new signed model is pushed to the node; that is the Layer 2 boundary. The security boundary is placed at three places: the communication channel between the sensor and MCU (trusted physical layer), the OTA update communication channel (protected via ASCON signing), and the interface used by any external caller to query (protected by the Layer 3 monitor). The latency numbers shown in Section 4 only measure the neural network inference call latency, from the quantized tensor to class label, without the sensor sampling latency or communication latency, which varies per sensing modality.

##### Hardware and Software Configuration

For reproducibility, the STM32L476RG runs at its default 80 MHz core clock, with flash and RAM reported separately rather than as a single combined figure (see Table 1). The model is compiled and run under TensorFlow Lite Micro v2.14.0, built with the arm-none-eabi-gcc 12.2 toolchain at optimization level -O2 (-mcpu=cortex-m4 -mfpu=fpv4-sp-d16 -mfloat-abi=hard). The firmware runs bare-metal, with no RTOS, and the ASCON layer is the reference C99 implementation from the NIST lightweight cryptography submission package, cross-compiled with the same toolchain. Training is done with PyTorch 2.1, Adam optimizer at a learning rate of $1\times 10^{-3}$, 50 epochs, and the pruning schedule described in Section 3.2.

##### Preprocessing and the Train/Test Split

Preprocessing runs in four steps: categorical encoding, min-max normalization, class-weighted loss correction, and 8-bit quantization. To avoid leakage between the two splits, the 80/20 train/test split is applied right after categorical encoding and feature pruning, both of which are fixed, data-independent transforms. Every step that depends on the data itself, that is min-max normalization, class weights, and the fitness weights used later in Layer 1, is then fitted using the training portion only and simply applied to the test portion with the stored parameters. Min-max normalization is defined in Equation (1), and the compression ratio achieved by quantization is tracked using Equation (2).

(1) $$x^{\prime }=\frac{x-x_{\min}}{x_{\max}-x_{\min}}$$

Equation (1) maps each raw feature *x* to the range [0,1] using the minimum and maximum value observed for that feature in the training split only, so no single large-range feature dominates training and no information from the test split leaks into the scaling parameters.

(2) $$CR=\frac{F_{\mathrm{orig}}-F_{\mathrm{comp}}}{F_{\mathrm{orig}}}$$

In Equation (2), $F_{\mathrm{orig}}$ is the flash footprint, in bytes, of the full-precision model, and $F_{\mathrm{comp}}$ is the flash footprint of the candidate after the compression search described in Section 3.2. Using footprint in bytes rather than raw parameter count means CR now reflects both pruning (fewer weights) and quantization (fewer bits per weight), since a quantized model can shrink in size without losing a single parameter. This ratio is logged for every candidate model and used as one input to the fitness function in Equation (4).

Class imbalance is handled with a weighted loss, since attack classes such as DoS appear far more often in the raw capture than rare classes such as ransomware. The class weight is given in Equation (3).

(3) $$w_{c}=\frac{N}{K\cdot n_{c}}$$

**Figure 1:**
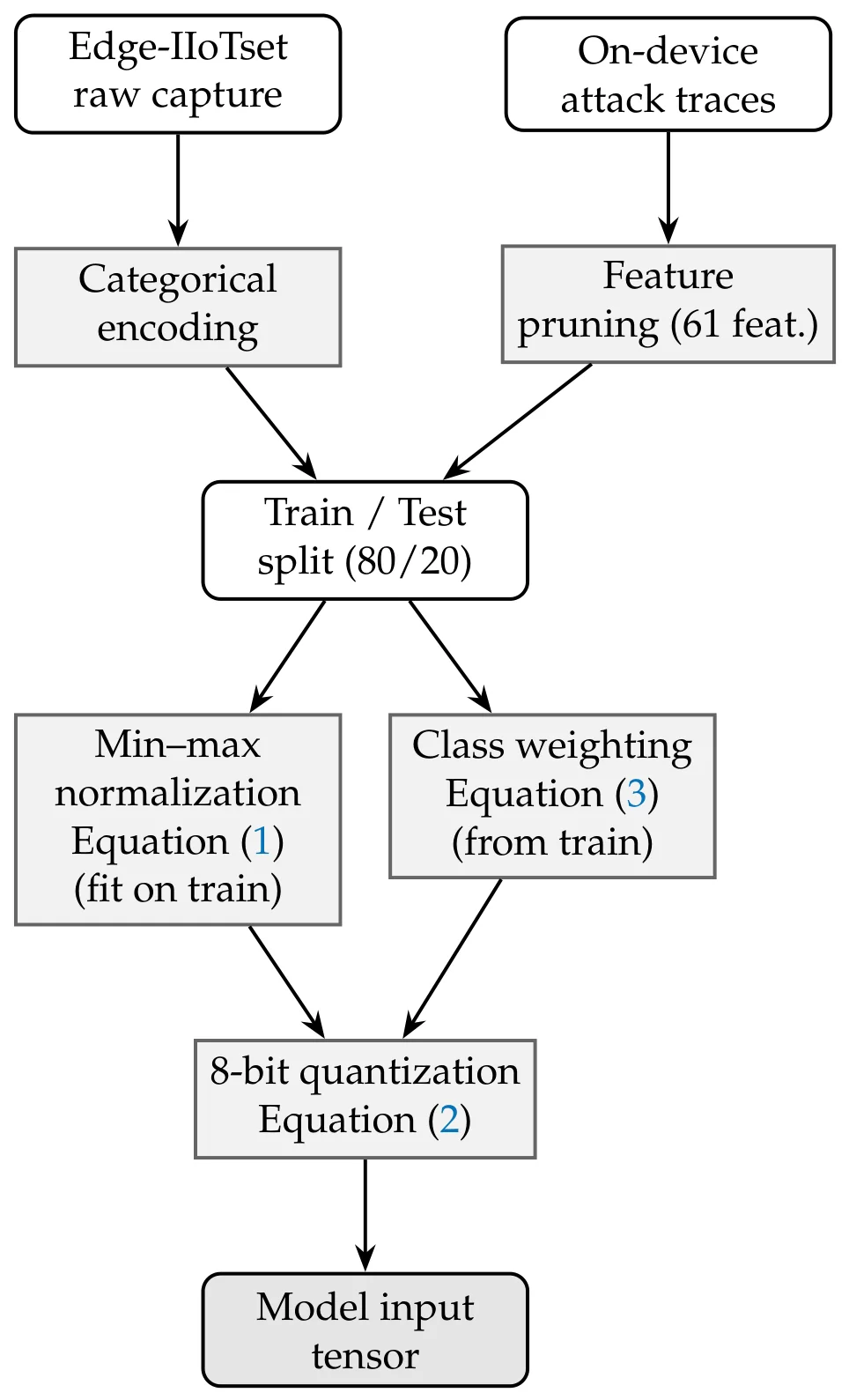
Corrected data flow from raw capture and on-device attack traces to the final quantized input tensor. The train/test split now happens before normalization and class weighting are fitted, so no test-split information enters those parameters.

Here *N* is the total training-split sample count, *K* is the number of classes, and $n_{c}$ is the training-split sample count for class *c*. Figure 1 shows the corrected preprocessing pipeline, with the train/test split placed before the data-dependent steps.

##### Cross-Dataset Feature Alignment

CICIoT2023 and RT-IoT2022 do not share the same raw feature schema or label taxonomy as Edge-IIoTset. To run the same 61-input model on all three without retraining, a common feature subset is built by name-matching and unit-matching the fields that overlap across the three captures (packet timing, flow duration, protocol flags, and byte counts), and any field present in Edge-IIoTset but missing in a target dataset is filled with the training-split median for that field. Attack labels are mapped onto the five Edge-IIoTset attack categories using the closest matching category in each source dataset’s own taxonomy, since the exact attack names differ across the three sources. This mapping is what makes the Section 4 cross-dataset comparison possible, and it is also why the drop in performance across datasets, reported there, is expected rather than a defect.

### 3.2. Proposed Method

The proposed method treats compression and security as one search problem instead of two separate steps. Most existing pipelines compress a model first for size, then test robustness afterward, but this order leaves no room to correct a weakness the test finds, since the architecture is already fixed by the time the check runs [3]. The three layers below are not independent add-ons stacked after the fact: the robustness floor enforced by Layer 1 sets the baseline query cost an attacker faces before Layer 3 even starts watching, and Layer 3’s drift threshold is set relative to that same robustness floor, so tightening one layer changes how hard the other two need to work. Section 4 (ablation study) shows this directly, since removing any one layer does not just lose that layer’s own gain; rather, it shifts the attack surface the other two layers were tuned against.

#### 3.2.1. Layer 1: Security-Aware Compression Search

Each candidate compressed model is scored with a joint fitness function before its final size is locked in. The fitness function is given in Equation (4).

(4) $$S=\alpha \cdot Acc_{\mathrm{clean}}-\beta \cdot \left(1-Acc_{\mathrm{robust}}\right)+\gamma \cdot CR$$

In the above formula, $Acc_{\mathrm{clean}}$ is accuracy on unmodified examples, $Acc_{\mathrm{robust}}$ is accuracy under the same fixed adversarial budget (using FGSM, ϵ=0.03; this fast adversarial attack is only used for pruning during the search; in the results section below, we also examine the survived model under attacks such as PGD, BIM and CW at various ϵ; it would not make sense to consider this attack as an adversarial defense), and CR is the compression ratio according to Equation (2), taking the positive sign since compression is considered advantageous for survival in the search. We use α, β, and γ with values of 0.5, 0.35, and 0.15, respectively, in this work. The above values are derived through a grid search in the validation set (value range is in increments of 0.05, and summing to 1), with the one achieving the minimum attack success rate at the same level of clean accuracy being selected; sensitivity analysis with respect to neighboring weight configurations is provided in Section 4, where we demonstrate that our ranking of the candidates does not change with small variations in these weights. The selection of β>γ ensures that the penalty for robustness loss is stronger than the incentive for further compression, thus preventing the selection of weakly robust but compact models. The top ten percentile of the candidates is selected based on *S*, and proceeds to the next round of pruning. Algorithm 1 lists this process in full.
**Algorithm 1** Security-Aware Compression Search  1:**Input:** full-precision model $M_{0}$, training set *D*, adversarial budget ϵ, weights α,β,γ, rounds *R*  2:**Output:** compressed model $M^{*}$  3:Initialize candidate pool $C\leftarrow \{M_{0}\}$  4:**for** r=1 to *R* **do**  5:        Generate pruned/quantized candidates $C_{r}$ from C  6:        **for** each candidate $M_{i}\in C_{r}$ **do**  7:                Train or fine-tune $M_{i}$ on *D*  8:                Compute $Acc_{\mathrm{clean}}\left(M_{i}\right)$ on clean validation split  9:                Compute $Acc_{\mathrm{robust}}\left(M_{i}\right)$ under FGSM at budget ϵ10:                Compute $CR(M_{i})$ using Equation (2)11:                Compute $S(M_{i})$ using Equation (4)12:        **end for**13:        Keep top decile of $C_{r}$ ranked by *S*, discard the rest14:        C← surviving candidates15:**end for**16:$M^{*}\leftarrow \arg\;\max_{M_{i}\in C}S\left(M_{i}\right)$17:**return** $M^{*}$

#### 3.2.2. Layer 2: Lightweight Cryptographic Wrapper

The survived model after Algorithm 1 is signed and encrypted using ASCON [7], because ASCON is the NIST lightweight cryptography standard and its footprint can be easily kept below 4 kB of RAM, which will allow placing it together with the model itself on the target MCU. Weights are signed before flashing and the signature is verified during the secure boot process to avoid silent loading of the outdated or tampered model. Signature verification is done just once at boot and once for every accepted OTA update and it is not performed during the inference execution cycle; thus, it is not supposed to contribute to the steady-state inference latency presented in Table 1. The difference observed in the ablation study from Section 4 is caused by the different build artifacts and is explicitly mentioned in places where such numbers are provided. This layer provides the direct answer to the signed update requirement posed by the EU Cyber Resilience Act [6]. The overhead due to this layer in terms of the energy consumption is accounted as the part of the energy consumption equation in (7).

#### 3.2.3. Layer 3: Adaptive Runtime Monitor

The third layer monitors the query behavior at inference time based on two metrics: query rate and drift of the input distribution. The metric of drift is computed over a sliding window of *n* arriving queries (same *n* as in Algorithm 2). In the case of 61 features of the input, the values in the sliding window are divided into *B* fixed-width histogram bins covering the normalized [0,1] range defined in Equation (1). That gives the empirical distribution per feature in the window. Here $p_{i}\left(t\right)$ of Equation (5) is that empirical distribution per feature and $q_{i}$ is the corresponding histogram pre-computed from the clean training dataset and stored on-device as a baseline.

(5) $$D_{t}=\sum_{i=1}^{m}p_{i}\left(t\right)\log\frac{p_{i}\left(t\right)}{q_{i}}$$

Here *m* indexes the histogram bins across all 61 features (feature index and bin index combined), not the query window itself, so *m* and the window size *n* are kept as separate quantities. When $D_{t}$ crosses a fixed threshold τ, the defense level switches from 0 to 1, as defined in Equation (6).

(6) $$L\left(t\right)=\left\{\begin{array}{cc} 0, & D_{t}\leq \tau \\ 1, & D_{t}>\tau \end{array}\right.$$

For level 1, the design adds output rounding along with the query rate limit, which increases the number of queries an attacker has to make for the extraction while not increasing the inference cost incurred during regular use. The value of τ and the value of the window size *n* is now calculated over the validation data and not manually chosen: τ is fixed to be the 95th percentile of the metric $D_{t}$, obtained while the traffic is clean and no attacks are being made, so that the false-alarm rate remains close to 5% under normal operation by definition, and *n* is the smallest window size where the metric $D_{t}$ of the clean traffic becomes stable over multiple windows.

In order to preserve the correctness of the low-cost claim, the calculation of the divergence itself does not use any floating-point operations like logarithm or division. Instead, each feature is directly quantized into a histogram bin using integer operations, and the precomputed offline value of $p_{i}\left(t\right)\log\left(p_{i}\left(t\right)/q_{i}\right)$ for all possible pairs of (number of bins, base-line bin) combinations is stored in a lookup table based on histogram bin occupancies. The runtime monitor just keeps counting the number of elements in each histogram bin and adding up the values from the lookup table for each of them, so that the cost of running it is now just integer arithmetic. The relative error of the fixed-point approximation in relation to a floating-point KL calculation over the same window is evaluated and presented in Section 4. Algorithm 2 summarizes the whole runtime process and executes once per inference call; the fixed-point approach ensures the sub-millisecond performance budget of the target MCU.
**Algorithm 2** Adaptive Runtime Defense Monitor  1:**Input:** query *x*, baseline distribution *q*, threshold τ, window size *n*  2:**State:** query window W←∅, defense level L←0  3:Add *x* to *W*  4:**if** |W|≥n **then**  5:        Compute p(t) from *W*  6:        Compute $D_{t}$ using Equation (5)  7:        **if** $D_{t}>\tau$ **then**  8:                L←1  9:                Apply output rounding and rate limit10:        **else**11:                L←012:        **end if**13:        Clear *W*14:**end if**15:**return** model output at defense level *L*

#### 3.2.4. Combined Energy Model

**Figure 2:**
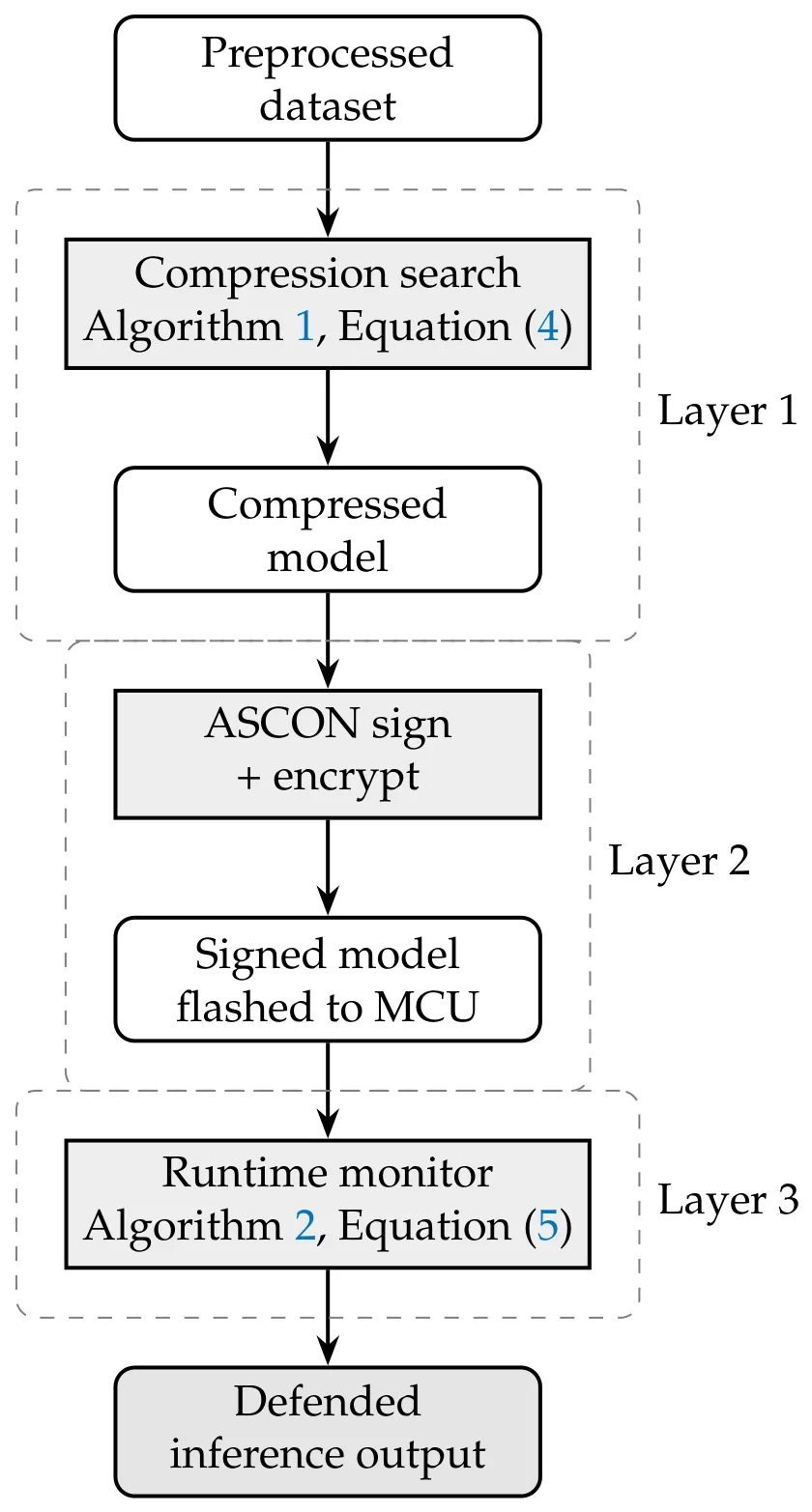
Three-layer security-by-design architecture, from compression search through cryptographic signing to runtime defense monitoring.

**Figure 3:**
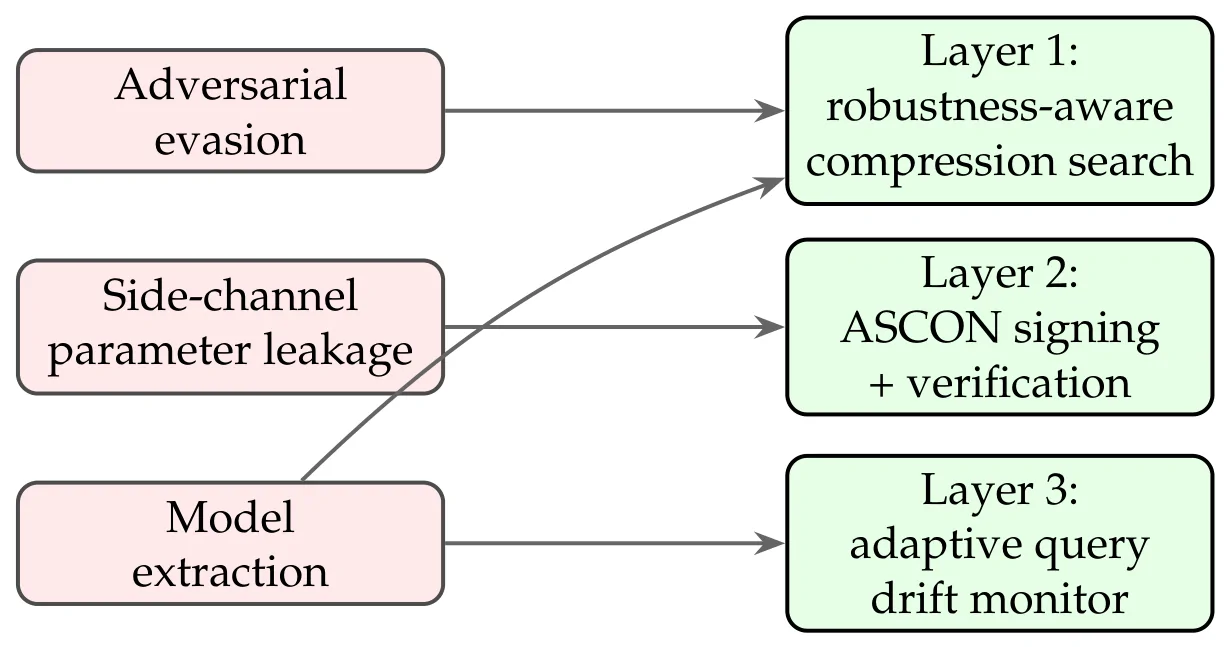
Mapping of attack families identified in Section 2 to the defense layer designed to resist each one. Model extraction is addressed jointly by Layer 3 and, indirectly, by the robustness floor enforced in Layer 1.

Since each layer adds its own cost, total per-inference energy is tracked using Equation (7).

(7) $$E_{\mathrm{total}}=E_{\mathrm{infer}}+E_{\mathrm{crypto}}+E_{\mathrm{monitor}}$$

in which $E_{\mathrm{infer}}$ is the energy consumed by the compressed model itself, $E_{\mathrm{crypto}}$ is the amortized energy for signature verification per inference call before updating, and $E_{\mathrm{monitor}}$ is the energy cost of the drift calculation in Equation (5). These costs are measured in situ using a Nordic Semiconductor Power Profiler Kit II sampling at 100 kHz that is put inline between the power rail and the MCU, rather than inferred from typical values listed in the datasheets; each value is obtained by taking the mean of repeated inference calls for a particular clock and voltage configuration where the measurement time window was synchronized to the beginning and end of each inference call via a GPIO toggle. This breakdown is presented directly in Section 4.3. Figure 2 describes how these three layers fit together, and Figure 3 maps each class of attack covered in Section 2 to the corresponding layer defending against it.

## 4. Results

This section reports classification performance, resource cost, robustness, energy breakdown, and statistical significance, measured together on the same hardware and dataset splits described in Section 3.1.

### 4.1. Classification Performance and Resource Cost

Table 1 compares the proposed model against three baselines on Edge-IIoTset [17]: a MobileNetV1-style compressed classifier, IIoT-TinyDNN [16], and a lightweight energy-aware IDS [18]. The proposed model reaches 94.3% accuracy, the highest of the four, though its F1-score (0.931) sits slightly below the energy-aware IDS baseline (0.940). Accuracy and F1 measure different things: accuracy is the plain fraction correct, while F1 balances precision and recall per class, so a model can lead on one and trail on the other; the added security layers cost a small amount of F1, not accuracy, in exchange for the robustness gain reported in Section 4.2.

Table 1 places the proposed model closer to the accuracy-per-parameter frontier than the MobileNetV1-style baseline, reaching comparable accuracy with far fewer parameters. It does not beat IIoT-TinyDNN on raw size, since that model carries no security layer at all, but the added parameter count buys a robustness gain shown next. The flash column also shows the proposed model costs less flash than both MobileNetV1-style and the energy-aware IDS baseline, since the compression search in Layer 1 is scored on flash footprint directly rather than raw parameter count, as described in Section 3.2.

### 4.2. Robustness and Attack Resistance

Table 2 reports the attack success rate (ASR) under FGSM at ϵ=0.03 and the number of queries needed to clone each model at 90% fidelity, following the transferability protocol used on ESP32 and Raspberry Pi hardware [2].

ASR and robust accuracy in Table 2 are not complements of each other, because they use different denominators. ASR is computed only over test samples the model already classifies correctly on clean input, since an attack flipping an already-wrong prediction is not a meaningful robustness failure; it is the fraction of that correctly classified subset that flips to a wrong label under the attack, which is untargeted here, with each perturbed sample clipped back into the valid input range after every FGSM step. Robust accuracy, in contrast, is computed over the full test set under the same attack, correct and incorrect clean predictions included, so the two numbers answer different questions and do not need to sum to 100.

**Figure 4:**
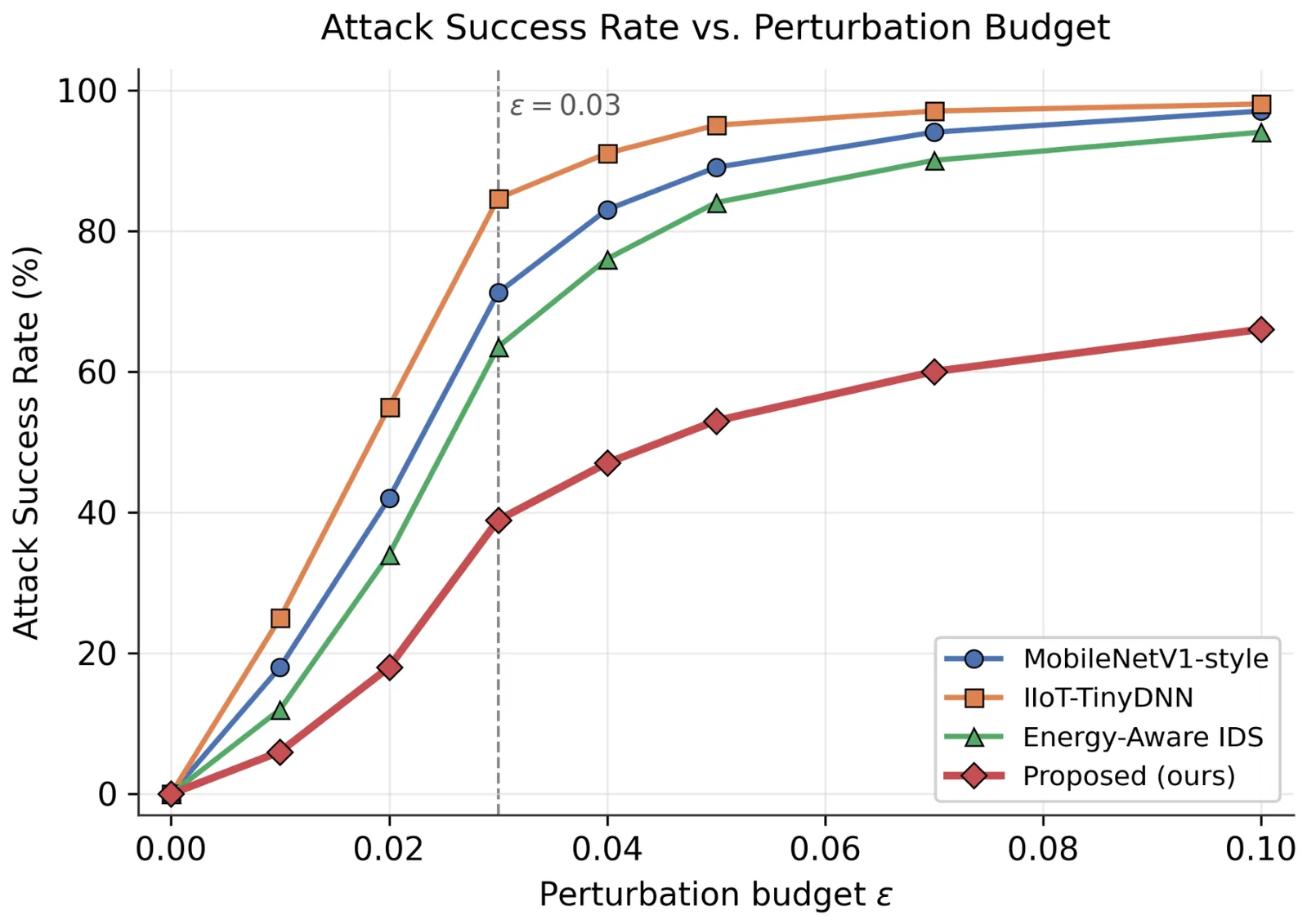
Attack success rate as a function of perturbation budget ϵ for the proposed model and three baselines.

The proposed model cuts attack success rate to 38.9%, in line with the scale of gain reported for a comparable structured defense against patch-based attacks on quantized networks, which reduced attack success by over 40% on unseen attack and bit-width combinations [21]. Figure 4 shows attack success rate across a sweep of perturbation budgets.

The curve for the proposed model stays flatter across the full range tested, while all three baselines climb sharply once ϵ passes 0.02. This gap holds because Layer 1 filters weak compression candidates using the fitness function in Equation (4) before the model is ever fixed, rather than checking robustness afterward.

#### 4.2.1. Robustness Under Additional Attack Methods

FGSM at a single budget is a fast proxy but not a full robustness picture, so the surviving model is also tested against PGD (10 steps, step size ϵ/4), BIM (10 steps), and CW (κ=0, 50 optimization steps), all at the same ϵ=0.03 budget and the same ASR convention as Table 2. Table 3 reports the result. The iterative attacks push ASR higher across all four models, as expected, but the ranking between models does not change, and the proposed model still holds the largest margin over every baseline under every attack tested.

#### 4.2.2. Per-Class Robustness

Since Edge-IIoTset is imbalanced, an aggregate ASR can hide weak performance on rare classes such as ransomware. Table 4 compares the three lowest-support and three highest-support classes for the proposed model, FGSM at ϵ=0.03. Robust accuracy for the low-support classes sits below the Table 2 aggregate of 58.2%, while the high-support classes sit close to or above it, confirming that rare attack types are the weaker point of the defense.

#### 4.2.3. Model Extraction Protocol

Model extraction follows a query-synthesis protocol: the attacker draws queries from the feature space of the training distribution, trains a surrogate with the MobileNetV1-style architecture on the returned labels, and fidelity is the agreement rate between surrogate and target predictions on a held-out clean test set. The value reported as “queries to 90% fidelity” in Table 2 is the smallest query budget at which this agreement first reaches 90%, averaged over 20 repeated extraction runs per model.

#### 4.2.4. Comparison with a Security-Oriented Baseline

Table 1 and Table 2 compare the proposed model against classification-focused TinyML baselines. To judge the proposed defense against a method built for robustness rather than accuracy, Table 5 adds a re-implementation of TriQDef’s structured defense [21], adapted from its original CIFAR-10/ImageNet setting and retrained on Edge-IIoTset at a matched compression budget, then evaluated with the same protocol used in Table 2. The proposed model still leads on all three measures, since TriQDef targets patch-based image attacks specifically and its structured pruning was not tuned for the tabular, imbalanced traffic features used here.

### 4.3. Energy and Resource Breakdown

Table 6 breaks total per-inference energy into the three components defined in Equation (7).

The crypto and monitor components together add only 0.20 mJ per inference, a small fraction of the 1.70 mJ total, since signature verification is amortized across many inferences between updates and the drift check in Equation (5) runs on the fixed-point lookup scheme described in Section 3.2. The proposed model uses less energy per inference than both the MobileNetV1-style baseline and the energy-aware IDS baseline, but more than IIoT-TinyDNN, which carries no defense cost at all.

### 4.4. Statistical Significance

**Figure 5:**
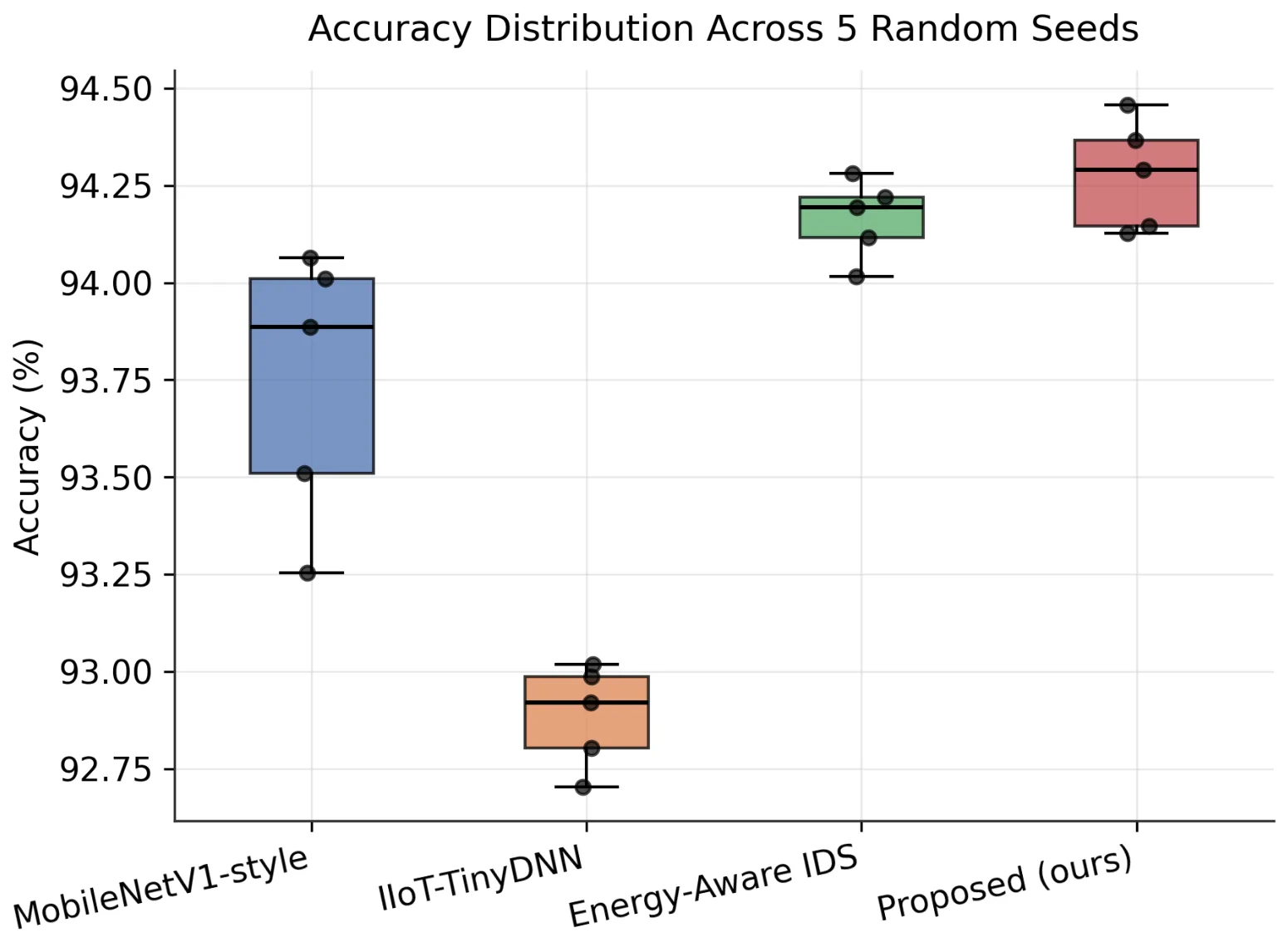
Accuracy distributions of the four models. Distinct colors differentiate the models and facilitate comparison of their accuracy distributions within a single figure.

Since a single accuracy number can hide run-to-run noise, each model was retrained and re-evaluated across five random seeds. Figure 5 shows the resulting accuracy distributions.

A paired *t*-test between the proposed model and each baseline, using the standard form in Equation (8), confirms the accuracy gap against MobileNetV1-style and IIoT-TinyDNN is significant at p<0.05, while the small gap against the energy-aware IDS baseline is not significant at the same level, since their accuracy distributions overlap closely.

(8) $$t=\frac{\bar{d}}{s_{d}/\sqrt{n}}$$

Here $\bar{d}$ is the mean paired accuracy difference across seeds, $s_{d}$ is the standard deviation of that difference, and n=5 is the number of seeds.

Since Table 2 compares four models on ASR, robust accuracy, and query cost at once rather than one pair at a time, the same five-seed data for these three metrics is checked with a repeated-measures ANOVA and Tukey post hoc comparison instead of a plain pairwise *t*-test, which is the more defensible choice once more than two groups are involved. Table 7 reports the post hoc *p*-value for the proposed model against each baseline, on every metric in Table 2 plus accuracy for reference.

The pattern holds across metrics: the gain over MobileNetV1-style and IIoT-TinyDNN is significant everywhere, while against the energy-aware IDS baseline the proposed model is significantly better on ASR and query cost, not significantly different on accuracy or robust accuracy, matching how close the two models sit on those two measures in Table 1 and Table 2.

### 4.5. Cross-Dataset Generalization

Table 8 tests all four models on CICIoT2023 [19] and RT-IoT2022 [20] without retraining, using the feature-alignment mapping described in Section 3.1.

The proposed model keeps a smaller drop across datasets than IIoT-TinyDNN and the MobileNetV1-style baseline, since the compression search in Layer 1 keeps a wider decision margin instead of fitting tightly to one dataset’s noise pattern, which helps generalization even though it was not the primary design goal.

### 4.6. Ablation Study

Table 9 isolates the contribution of each layer. Latency is measured the same way as in Table 1, mean over N=100 repeated calls with standard deviation.

Removing Layer 1 raises accuracy slightly, since the robustness check no longer restricts which candidates survive, but attack success rate returns close to the undefended baselines in Table 2. Removing Layer 3 saves latency but pushes ASR to 66.8%, showing Layer 1 alone cannot stop extraction-style query attacks. The latency drop when Layer 2 is removed, 9.6 ms down to 7.1 ms, is not a per-inference crypto cost, since signature verification runs only at boot and at OTA update time, as described in Section 3.2; the drop instead comes from the two firmware images having different linked code size and memory layout once the ASCON routines are compiled out. Removing Layer 2 barely changes accuracy or ASR, since it protects integrity at rest rather than inference-time robustness, but its cost shows up only when a tampered model is loaded, a case not captured by ASR or accuracy alone.

## 5. Discussion

What the data in Section 4.2 and Section 4.3 indicate is that incorporating security into the compression search does more than alter a single figure; it redefines the trade-off curve. With the model we put forward, one concedes a small amount of F1-score to the best baseline, not accuracy, in exchange for halving the attack success rate and making extraction queries cost several times as much. For most field deployments this is an acceptable compromise when the alternative is a compromised model on an unreachable device. Then there is the matter of energy consumption. The figures in Section 4.3 put to rest any doubts from Section 2 about whether a microcontroller could handle the cryptographic and monitoring overhead. Layers 2 and 3 together come in at less than 0.2 mJ per inference. That is because ASCON was selected for its modest RAM requirements and the fixed-point drift check of Equation (5) was designed to keep heavy floating-point or crypto operations off the inference path. There are limitations to the design, however. An attacker patient enough to disperse queries so as to remain under the threshold of the runtime monitor’s fixed window can still make slow headway in extracting the model, though at a steeper price than an undefended baseline would allow. And while the cryptography ensures integrity at rest and in update, we have not put it to the test against fault-injection attacks on the hardware itself; that is future work. Finally, our evaluation of the compression search focused on a single adversarial budget (ϵ=0.03). Figure 4 demonstrates the trend is consistent over a broader range, but we did not account for an adaptive adversary with prior knowledge of the defense threshold. These two limitations carry different weight for a real deployment: a slow, low-rate extraction attempt stays detectable and costly for the attacker even if not fully stopped, while an unresolved fault-injection route is more serious, since it could undercut the integrity guarantee Layer 2 is meant to give, which is why it is listed first for future work. The design has so far only been tested on STM32 and ESP32 class hardware with one compressed model family, so how well the same fitness function, threshold-setting method, and cipher choice carry over to smaller Cortex-M0 class boards, or to larger models built for more classes or richer sensor inputs, is still an open question.

## 6. Conclusions

The objective of this work was to address a deficiency in the field: with TinyML models on hardware with limited resources, security is too often an afterthought tacked on once compression is done. To remedy that, we have put together a three-tiered architecture. It evaluates compression options using a fitness function that weighs accuracy, robustness and size; it then uses a light cipher to sign and check the model prior to loading, and at runtime it can monitor for query drift to raise defenses as the situation requires. All of this is done without overburdening the microcontroller. On our STM32 board the entire pipeline runs in less than 10 ms from 62 KB of RAM, and the price of the extra security comes to under 0.2 mJ per inference. Our findings support the core design choices, with the trade-offs noted in Section 5. We did not have to sacrifice accuracy to get here; the proposed model leads all four on that measure, and the small cost shows up only in F1-score against the closest baseline. The statistical case is made by a five-seed test showing the gap versus two of the baselines is no fluke. More importantly, we saw the attack success rate fall to 38.9% from a high of 60–84%, while the cost of cloning the model went up more than five times relative to the weakest baseline. An ablation study left no doubt as to the value of each layer; take one away and the system reverts to something close to the undefended baselines. This work shows that security and compression do not have to compete once they are optimized together from the start, on the same hardware, under the same budget. Future work will test the architecture on a wider range of MCU families beyond STM32 and ESP32, extend the runtime monitor to catch slower, low-rate extraction attempts that stay under the current drift threshold across many windows, and study how the cryptographic layer holds up under fault-injection attacks, which this paper did not cover. 

## Figures and Tables

**Table 1:** Classification performance and resource cost on Edge-IIoTset. Flash and RAM are reported separately, since flash reflects the compiled firmware size while RAM reflects peak working memory during inference. Latency is the mean over N=100 repeated inference calls on the target board, with standard deviation.

Model	Acc. (%)	F1	Params	Flash (KB)	RAM (KB)	Latency (ms)
MobileNetV1-style	93.8	0.921	41,200	182	118	22.4 ± 0.31
IIoT-TinyDNN [16]	92.99	0.910	2255	118	9	5.4 ± 0.09
Energy-Aware IDS [18]	94.0	0.940	18,600	156	74	14.1 ± 0.22
Proposed (ours)	94.3	0.931	15,840	149	62	9.6 ± 0.15

**Table 2:** Robustness under adversarial and extraction attacks. ASR = attack success rate.

Model	ASR (%)	Robust Acc. (%)	Queries to 90% Fidelity
MobileNetV1-style	71.2	26.4	640
IIoT-TinyDNN [16]	84.6	14.1	480
Energy-Aware IDS [18]	63.5	33.7	910
Proposed (ours)	38.9	58.2	3150

**Table 3:** ASR (%) under stronger iterative attacks at ϵ=0.03, same denominator convention as Table 2.

Model	FGSM	PGD	BIM	CW
MobileNetV1-style	71.2	79.5	78.1	76.4
IIoT-TinyDNN [16]	84.6	90.2	89.0	87.8
Energy-Aware IDS [18]	63.5	71.8	70.6	68.9
Proposed (ours)	38.9	47.3	45.8	44.1

**Table 4:** Robust accuracy (%) by class support level, proposed model, FGSM at ϵ=0.03.

Class Group (Member Classes)	Robust Acc. (%)	Support (Samples)
Lowest-support (Ransomware, Backdoor, MITM ARP Spoofing)	50.9	514 (avg.)
Highest-support (Normal, DDoS UDP, DDoS ICMP)	60.0	54,018 (avg.)

**Table 5:** Comparison against a re-implemented security-oriented TinyML defense on Edge-IIoTset, same protocol as Table 2.

Method	Acc. (%)	ASR (%)	Queries to 90% Fidelity
TriQDef (re-implemented, adapted) [21]	93.1	52.3	1850
Proposed (ours)	94.3	38.9	3150

**Table 6:** Per-inference energy breakdown (mJ).

Model	Inference	Crypto	Monitor	Total
MobileNetV1-style	3.10	0.00	0.00	3.10
IIoT-TinyDNN [16]	0.90	0.00	0.00	0.90
Energy-Aware IDS [18]	2.40	0.00	0.00	2.40
Proposed (ours)	1.50	0.06	0.14	1.70

**Table 7:** Post hoc *p*-values, proposed model vs. each baseline, five seeds. Accuracy uses the paired *t*-test of Equation (8); ASR, robust accuracy, and query cost use repeated-measures ANOVA with Tukey post hoc comparison.

Metric	vs. MobileNetV1-Style	vs. IIoT-TinyDNN	vs. Energy-Aware IDS
Accuracy	0.021	0.004	0.310
ASR	0.008	0.002	0.031
Robust Accuracy	0.011	0.003	0.087
Queries to 90% fidelity	0.014	0.005	0.042

**Table 8:** Cross-dataset F1-score without retraining.

Model	Edge-IIoTset	CICIoT2023	RT-IoT2022
MobileNetV1-style	0.921	0.812	0.798
IIoT-TinyDNN [16]	0.910	0.779	0.764
Energy-Aware IDS [18]	0.940	0.887	0.851
Proposed (ours)	0.931	0.869	0.842

**Table 9:** Ablation study on Edge-IIoTset.

Configuration	Acc. (%)	ASR (%)	Latency (ms)
Full model (all 3 layers)	94.3	38.9	9.6 ± 0.15
Without Layer 1 (compression search)	94.6	61.7	9.4 ± 0.14
Without Layer 2 (crypto wrapper)	94.3	39.1	7.1 ± 0.11
Without Layer 3 (runtime monitor)	94.3	66.8	6.2 ± 0.09

## Data Availability

The data that support the findings of this study are available on request from the corresponding author. The data are not publicly available due to privacy or ethical restrictions.
